# Advances in the Treatment of Neuropathic Pain by Sympathetic Regulation

**DOI:** 10.1007/s11916-024-01285-9

**Published:** 2024-06-22

**Authors:** Ping Xu, Han Rui Fan, En Ming Zhang, Hao Nan Zhang, Yong Fei

**Affiliations:** 1 Zhejiang Chinese Medical University, Hangzhou, Zhejiang People’s Republic of China; 2 Department of Anesthesiology and Pain, The First Hospital of Jiaxing, Jiaxing, Zhejiang People’s Republic of China; 3 Jiaxing University, Jiaxing, Zhejiang People’s Republic of China

**Keywords:** Stellate ganglion block, Neuropathic pain, Sympathetic regulation, Sympathetic radiofrequency thermocoagulation

## Abstract

**Purpose of Review:**

To explore the mechanism and therapeutic effect of sympathetic nerve regulation on neuropathic pain.

**Recent Findings:**

A comprehensive search was conducted in the PubMed and CNKI libraries, using the following keywords: stele ganglion block, neuropathic pain, sympathetic nerve block, sympathetic chemical destruction, and sympathetic radiofrequency thermocoagulation. We selected and critically reviewed research articles published in English that were related to sympathetic modulation in the treatment of neuropathic pain. The collected literature will be classified according to content and reviewed in combination with experimental results and clinical cases. Neuropathic pain was effectively treated with sympathetic regulation technology. Its mechanism includes the inhibition of sympathetic nerve activity, regulation of the inflammatory response, and inhibition of pain transmission, which greatly alleviates neuropathic pain in patients. Stellate ganglion blocks, thoracic and lumbar sympathectomies, chemical destruction, and radiofrequency thermocoagulation have been widely used to treat neuropathic pain.

**Summary:**

Sympathetic regulation can effectively relieve pain symptoms and improve the patient's quality of life by inhibiting sympathetic nerve activity, reducing the production and release of pain-related mediators, and inhibiting pain transmission. CT-guided radiofrequency thermocoagulation of the thoracic and lumbar sympathetic nerves is effective and durable, with few complications, and is recommended as a treatment for intractable neuropathic pain.

## Introduction

Neuropathic pain is defined as pain caused by lesions or diseases of the somatosensory nervous system [1–3], It is often a complex syndrome caused by a variety of diseases or injuries and manifests as a combination of multiple symptoms and signs. Pain symptoms are primarily divided into non-stimulus-dependent and stimulus-dependent types. Non-stimulus-dependent pain types include persistent and spontaneous pain. The stimulus-dependent types include hyperalgesia and dysalgesia. Patients often experience burning pain, shooting pain, tingling pain, dysodynamic mechanical pain, and numbness [4–6]. In addition, loss of sensation in areas of pain and dysfunction of the autonomic nervous system of the skin may also occur. The impact of chronic neuropathic pain on an individual can be overwhelming, and it can severely disrupt daily activities, lead to decreased quality of life, decrease sleep and appetite, and even induce depression and anxiety [7–9]. Common neuropathic pain-related diseases include trigeminal neuralgia, postherpetic neuralgia, diabetic peripheral neuropathy, complex regional pain syndrome, post-stroke pain, cancer-related neuropathic pain, and various neuralgia after surgery [10–13]. Traditional treatment methods include drug therapy, physical therapy, such as exercise and rehabilitation, and psychological therapy such as psychological counseling; however, often cannot fundamentally relieve patients' pain symptoms [14–16]. With the development of neuroregulatory techniques, spinal cord electrical stimulation and dorsal root ganglion pulsed radiofrequency are commonly used to treat neuropathic pain [17, 18]. Although SCS is effective in the treatment of neuropathic pain, owing to the implantation of electrodes into the spinal canal, the risk of epidural infection is positively correlated with the implantation time, and there is a risk of electrode displacement or even fracture. In addition, patients need to pay tens of thousands of yuan as out-of-pocket medical expenses, which limits their clinical application [19]. The short-term effect of dorsal root ganglion PRF is accurate. Puncture needles are the most commonly used minimally invasive means for the treatment of neuropathic pain because they are safer and cheaper than SCS and do not enter the spinal canal. However, poor long-term efficacy remains a major drawback. To alleviate pain in patients with neuropathic pain, clinicians have been exploring various effective treatment methods, such as star ganglion block, sympathetic nerve chemical destruction, sympathetic nerve RF thermocoagulation, and dorsal root ganglion pulse RF (PRF) combined with sympathetic nerve RF. This review aimed to integrate the literature on sympathetic regulation of neuropathic pain from the National Library of Medicine (PubMed) and China National Knowledge Network (CNKI) databases from January 1, 2000, to December 1, 2023, and provide an overview of the above methods.

## The Anatomical Basis of Sympathetic Nerve

The sympathetic nervous system is part of the visceral nervous system, also known as the autonomic nervous system (ANS). Anatomically, there is a paraspinal sympathetic trunk on the left and right sides of the spinal cord, which is connected to the ventral branch of the spinal nerve, from the base of the skull to the tailbone. In front of the coccyx, the two branches merge into odd ganglia, whose postganglionic fibers enter the spinal nerve through the grey communicating branch and are distributed in the surrounding and internal organs. The thoracic sympathetic trunk consists of 12 thoracic ganglia that innervate the aortic, cardiac, and pulmonary plexuses, as well as the abdominal organs through the abdominal, upper, and lower mesenteric plexuses. The lumbar sympathetic nerve trunk is located in the anterolateral part of the lumbar vertebral body and the anterior part of the psoas major muscle. The right trunk is located behind the lateral edge of the inferior vena cava, and the left trunk is located at the lateral edge of the abdominal aorta. There are 2–6 pairs of lumbar sympathetic ganglia, most of which are located at the level of the corresponding vertebral body or between the upper and lower vertebral bodies, and are usually located in the lateral nucleus of the lateral column of the spinal gray matter at the third position of the lumbar spine. The main areas of innervation include the lower limbs, buttocks, and the ischium [20–22].

## The Principle and Mechanism of Sympathetic Regulation Therapy

The mechanism of sympathetic regulation is mainly to regulate vasomotor function and pain transmission. Firstly, the sympathetic nervous system is involved in vasomotor regulation. When the sympathetic nerve is excited, it causes peripheral vascular contraction, resulting in a relative decrease in blood flow and insufficient blood supply. This increases the anaerobic metabolism of the muscle, producing metabolites such as lactic acid, and eventually causing pain. When the sympathetic nerve is damaged by mechanical, chemical, or high temperature, the sympathetic nerve tension decreases, resulting in relative vasodilation, reduced peripheral vascular resistance, increased collateral and peripheral circulating blood volume, and increased blood perfusion in skin and muscle of extremities through "loss of sympathetic effect" [23–25]. Secondly, the sympathetic nerve is involved in the production and release of pain-related mediators, such as nerve growth factor, interleukin-8, bradykinin, calcitonin gene-related peptide, and substance P [26]. After the sympathetic nerve is blocked or destroyed, pain stimulation can be blocked to the central nervous system through nerve fibers, and the regeneration of skin vascular cells can be regulated by inhibiting the proliferation of parietal cells and increasing the expression of angiopoietin-1, reducing the inflammatory response in the denervation area of the sympathetic nerve and reducing the release of adrenergic energy in the dorsal root ganglion. By stimulating and/or upregulating α2-adrenergic receptor, the sympathetic nerve activity is inhibited, the activation of spinal microglia is inhibited, and the expression of inflammatory factors (IL-1β, IL-6, and TNF-α) is decreased, thus inhibiting pain transmission, and achieving the purpose of relieving neuropathic pain [27–29]. Therefore, sympathetic regulation plays an important role in the treatment of neuropathic pain [30].

## Sympathetic Nerve Regulation Therapy

**Fig. 1:**
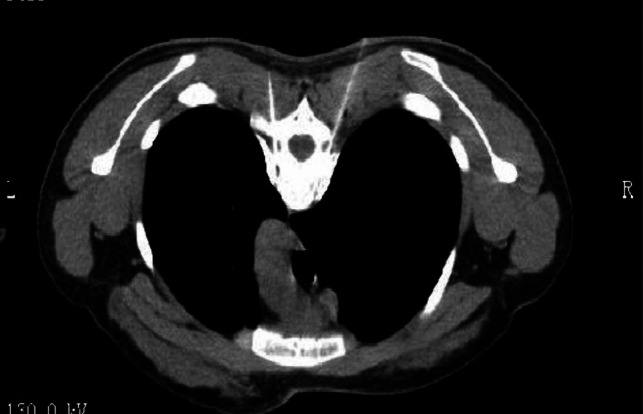
Thoracic sympathetic anhydrous alcohol block(T4)

1. stellate ganglion block (SGB)
  The stellate ganglion, also known as the cervicothoracic ganglion, is usually formed by a combination of the lower cervical and T1 ganglions. It is located in front of the transverse process of the C7 vertebral body, directly below the subclavian artery. The postganglionic fibers are connected to eight pairs of cervical nerves via the gray communication branch and distributed along the cervical nerves to the blood vessels, sweat glands, and trichorectus of the head and neck and upper limbs. It also branches into neighboring arteries, forming the internal carotid plexus, external carotid plexus, subclavian plexus, vertebral plexus, and pharyngeal plexus, and enters the thoracic cavity to participate in the formation of the cardiac plexus [31, 32]. Therefore, stellate ganglion block has been widely used in the treatment of various vascular diseases with sympathetic nerve-mediated pain in the upper limb, head and neck, such as trigeminal neuralgia, chronic migraine, complex regional pain syndrome (CRPS), cephalic and facial postherpetic neuralgia [33–37]. For rare thalamic pain, a type of central pain after stroke, which is usually unbearable and uncontrollable and seriously affects the quality of life of patients, stellate ganglion block, as an effective treatment for sympathetic nerve regulation, can be an alternative treatment for thalamic pain after stroke. Especially when pain is associated with autonomic nervous changes, such as mood changes, ultrasound-guided SGB can also be used in the treatment of patients with thalamic pain syndrome (TPS) caused by thalamic cancer, before more invasive intracranial surgery is performed to treat pain [38, 39]. The stellate ganglion block is a procedure in which a local anesthetic is injected into and around the stellate ganglion to temporarily block the sympathetic nerve, thereby improving the blood supply to the head and neck, inhibiting overexcitation of the sympathetic nerve, and relieving pain [40]. The stellate ganglion block operation is simple, and the presence of Horner syndrome after the block can be regarded as a sign of a successful block. Lopes and Fischer [41]. reported that a procain stellate ganglion block successfully treated a patient with severe trigeminal neuralgia. After ultrasound and other imaging guidance, the accuracy and safety of the block were further improved. Yu et al [42]. reported that an ultrasound-guided stellate ganglion block is an effective treatment for chronic migraines.
  The exact mechanism underlying the analgesic effect of SGB on sympathetic maintenance pain remains unclear. Owing to the occurrence of long-term Horner syndrome, it is not appropriate to perform a detrusive block or radiofrequency thermocoagulation for a staphyloid ganglion block. Due to the short duration of the postoperative curative effect of the staphyloid ganglion block, the postoperative recurrence rate may be significantly increased, increasing the pain of patients, and multiple block therapy is often required.
2. Endoscopically assisted sympathectomy
  Thoracoscopic thoracic sympathectomy or retrolaparoscopic lumbar sympathectomy can completely remove the target sympathetic nerve [43], while the sympathetic effect of the corresponding sympathetic innervation area is lost and other innervation areas are relatively active. Complications, such as hemopneumothorax, postoperative pulmonary infection, atelectasis, pulmonary edema, intercostal neuralgia, accidental peritoneal tears, muscle hematoma, pneumoperitoneum, incision infection, and sexual dysfunction, may occur during endoscopic sympathectomies. Therefore, the operation needs to be completed under general anesthesia, and the anesthesia and surgical trauma are relatively large. Postoperative compensatory hyperhidrosis was the primary cause of patient dissatisfaction [44, 45]. Compensatory hyperhidrosis rarely improves over time and can occur anywhere, with the thighs and groins being most commonly affected. Studies have shown that the higher the level of sympathetic chain transection (especially the T2 level), the greater the risk of compensatory sweating.
3. Chemical thoracic sympathetic Block
  A thoracic sympathetic block can be performed under local anesthesia with minimal trauma. Guided by CT, the tip of the puncture needle was placed above the small head of the ribs at T3 or T4, and 2 mL of 1% lidocaine containing the contrast agent iodohexol was injected next to the thoracic sympathetic nerve. The perfusion index (PI) of the small fingers of both hands increased by more than five times and the temperature of the palms of both hands increased by more than 3 ℃. That is, 2.5 ml of anhydrous ethanol containing the contrast agent iodohexol was injected into each side after 20 min (Fig. 1). CT scan was repeated and three-dimensional reconstruction was performed to observe the distribution of the drug solution. The drug solution covered the small heads of the ribs at T3 and T4 (Fig. 2) [46–48]. Anhydrous ethanol (AE) is a commonly used drug for chemical damage. Due to the poor controllability of anhydrous ethanol, serious complications may occur during clinical operations, such as anhydrous ethanol entering blood vessels, resulting in spinal artery embolism and paraplegia. In the case of chemical destruction of the thoracic sympathetic nerve (T3 is more common), anhydrous ethanol can diffuse up to the first rib, resulting in permanent Horner syndrome, although this can be corrected by diluting the stele with a saline injection of anhydrous ethanol, again with a reduced clinical effect. In addition, diffusion of anhydrous ethanol can damage the intercostal nerves. Due to the risk and poor efficacy of uncontrollable anhydrous ethanol, radiofrequency ablation of the thoracic sympathetic nerve has been used clinically to replace chemical destruction [49].

**Fig. 2:**
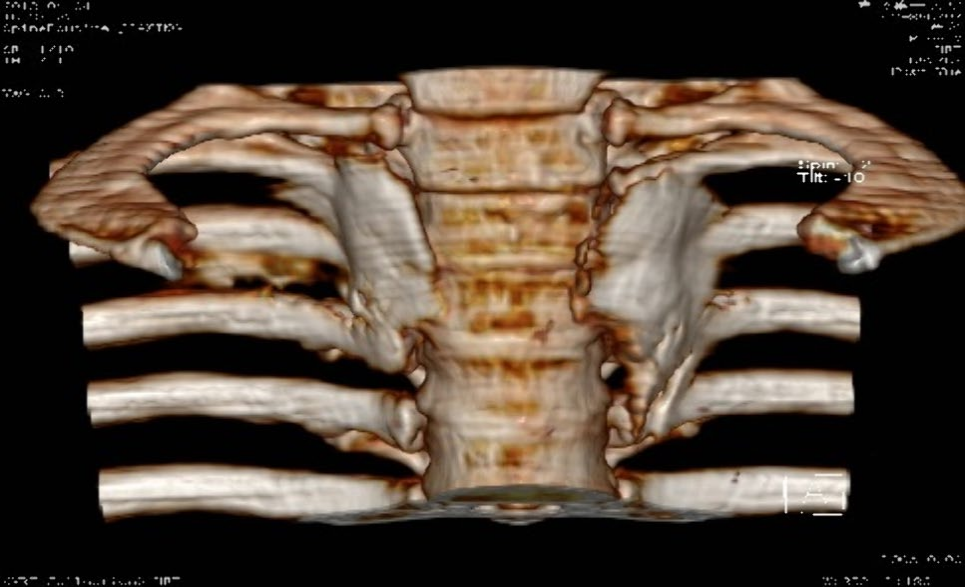
Thoracic sympathetic anhydrous alcohol block(T4)—Three-dimensional reconstruction
4. Chemical lumbar sympathetic nerve block
  The lumbar sympathetic nerve block can also be performed under local anesthesia [50]. The tip of the puncture needle was guided by CT to the anterolateral part of the L3 vertebral body and the anterior part of the psoas major muscle, and 3 mL of 1% lidocaine containing the contrast agent iodohyl was injected into the lumbar sympathetic nerve. It was observed on the CT scan that the injected liquid in the lumbar segment was distributed between the psoas major muscles on both sides and the L2 and/or L3 vertebral body (Fig. 3). When the PI of the patient's toes increased by more than five times and the temperature of the soles of the feet increased by more than 3℃, 5 m of anhydrous ethanol containing the contrast agent iodihexyl alcohol was injected into the lumbar puncture site. CT scan was repeated, and a three-dimensional reconstruction was performed to observe the distribution of the drug solution (Fig. 4). The operation was completed when the drug solution covered the anterior sides of the L2 and L3 vertebrae. During lumbar sympathetic neurochemical destruction (L2 is more common), anhydrous ethanol diffuses up to the lumbar 1 level, leading to ejaculation disorders. In addition, the diffusion of anhydrous ethanol can damage the sciatic nerve and even lead to permanent damage to the lateral femoral cutaneous nerve [51].

**Fig. 3:**
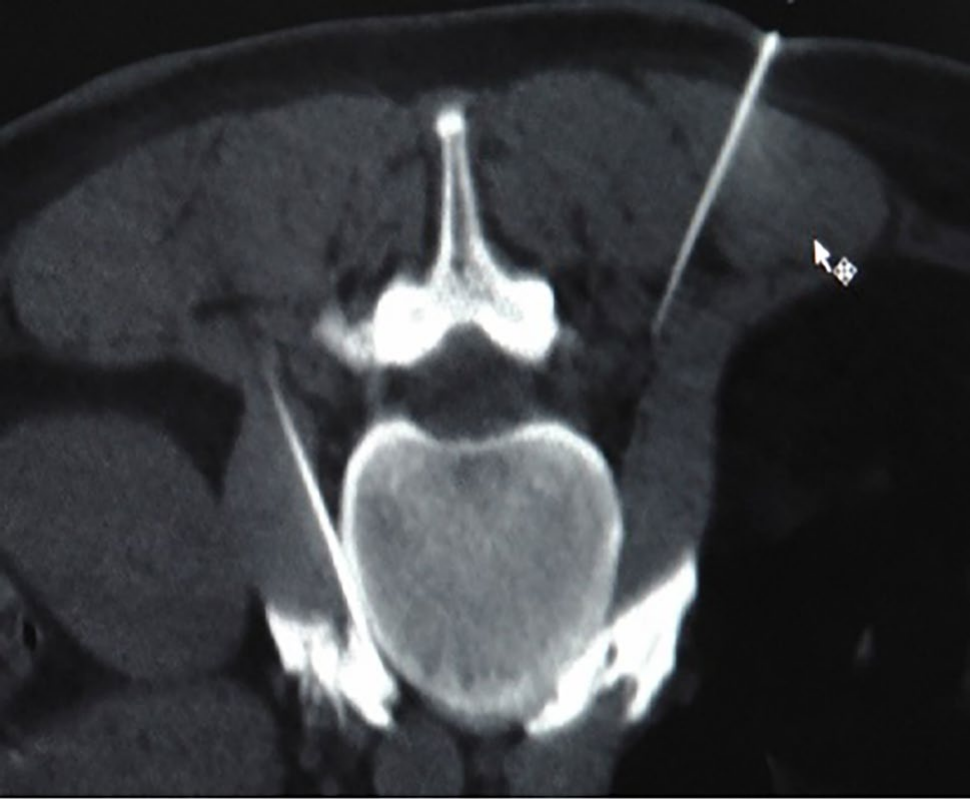
Lumbar sympathetic nerve anhydrous alcohol block(L3)

**Fig. 4:**
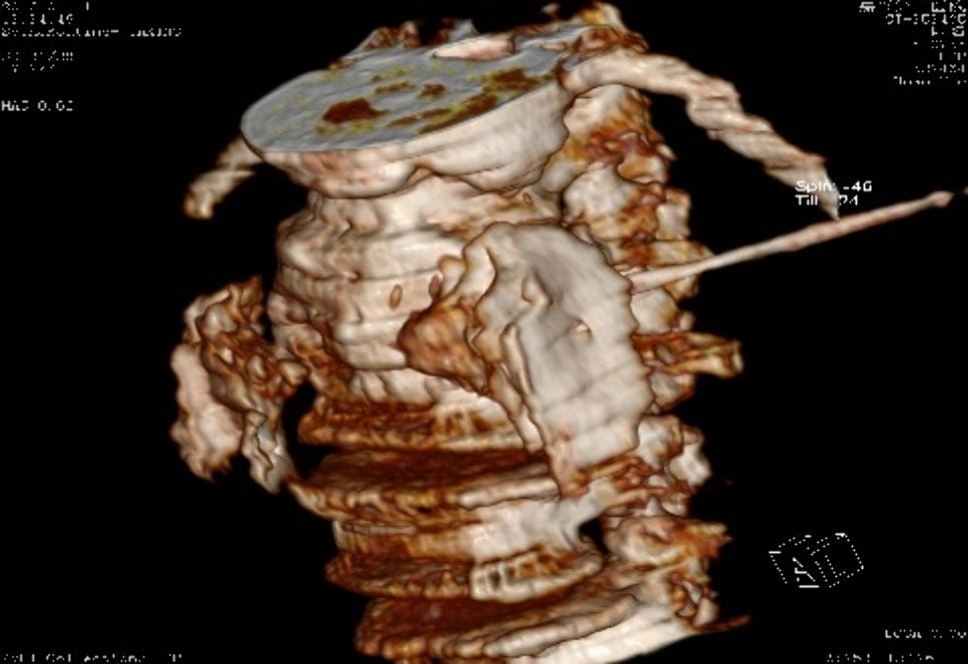
Lumbar sympathetic nerve anhydrous alcohol block(L3)—Three-dimensional reconstruction
  Primary erythematous melalgia (PE) is a rare pain disorder characterized by severe burning pain, erythema, and fever in the limbs. Ogawa et al. [52] successfully treated a case of PE using a lumbar sympathetic ganglion block (LSGB) combined with an epidural block. Complex regional pain syndrome (CRPS) is a typical neuropathic pain disease. Fractures, trauma, and surgery are common inducers of CRPS, and pain is often persistent and difficult to cure [53]. Studies have shown that chemical sympathetic regulation can destroy the chemical coupling between the postganglionic fibers of the sympathetic nerve and primary afferent neurons and restore normal conduction function to relieve pain. Abramov [54] studied 29 patients with CRPS after knee surgery and found that 85% had partial or complete relief of knee pain after several lumbar sympathetic nerve block treatments before and after surgery. Shoji et al. [55]. reported that lumbar sympathetic nerve block (LSNB) improved lower limb blood flow, increased skin temperature, increased skin perfusion pressure, and reduced the pain of sympathetic afferents by promoting lumbar sympathetic ganglion block for wound healing in a rat posterior limb ischemia model through basic research. Recently, a new technology, laser speckle flow imaging (LSFG), has achieved non-invasive quantitative and qualitative blood flow assessment for some peripheral artery diseases lacking lower extremity skin temperature changes and has confirmed the effectiveness of sympathetic nerve block [56].
  Chemical sympathetic nerve block has a certain effect in the treatment of neuropathic pain, but the blocker has a certain fluidity and relatively poor controllability, which not only causes serious complications but also reduces efficacy and patient satisfaction. The chemical sympathetic block technique has been gradually replaced with sympathetic radiofrequency ablation.
5. Radiofrequency thermocoagulation of thoracic sympathetic nerve
  Radiofrequency thermocoagulation of the thoracic sympathetic nerve was the original technique used by our research team. Taking T4 sympathetic radiofrequency as an example, the surgical steps were as follows: the patient was lying prone on the CT table, and the vital signs, pulse oxygen saturation, peripheral perfusion index, and palm temperature of the patient were monitored in real-time using a multifunctional vital detector. The T3-4 vertebral space on the affected side was located on the CT. After a plain CT scan, the optimal puncture level and puncture point on the skin were selected, and the depth, angle, and distance from the midline of the injection point were simulated. After local invasive anesthesia was completed at the selected puncture site, the needle was inserted through the T3-4 paravertebral space across the costotransverse joint to the posterolateral head margin of the T4 ribs of the vertebral body (Fig. 5), and the resistance of the surrounding tissue from the tip of the needle to the tip of the test electrode was adjusted to 250-550Ω. After sensory and motor electrical stimulation tests were performed to confirm the correct position of the RF tip, First, 40 ℃ was set to give thermal stimulation for 60 s, and then the temperature was set to 95 ℃. The procedure was completed after the thermal coagulation, which lasted for 300 s. The 3D reconstruction clearly showed that the puncture needle was placed on the outside of the small head of the rib (Fig. 6).

**Fig. 5:**
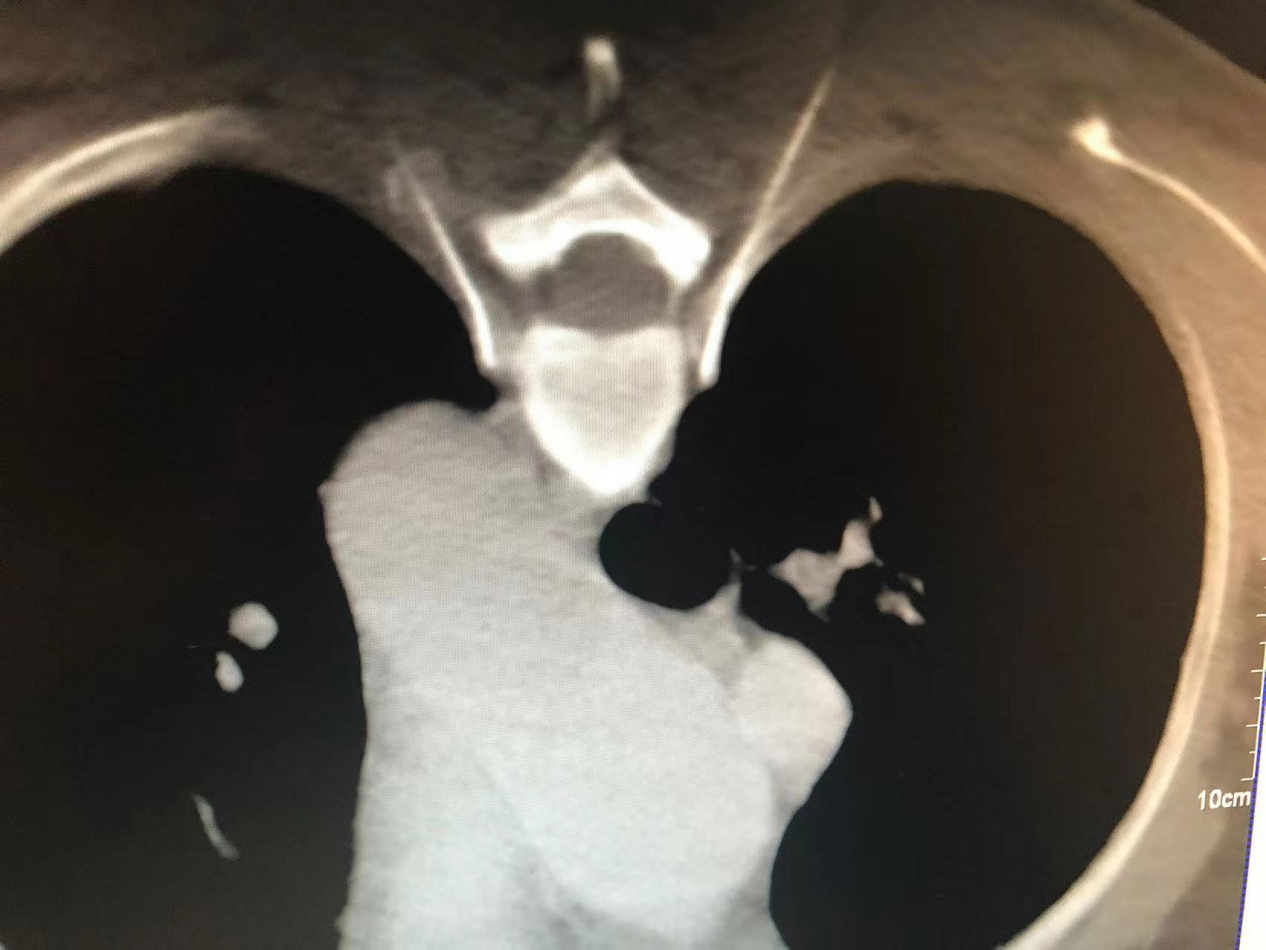
Radiofrequency thermocoagulation of bilateral thoracic sympathetic nerve(T4)

**Fig. 6:**
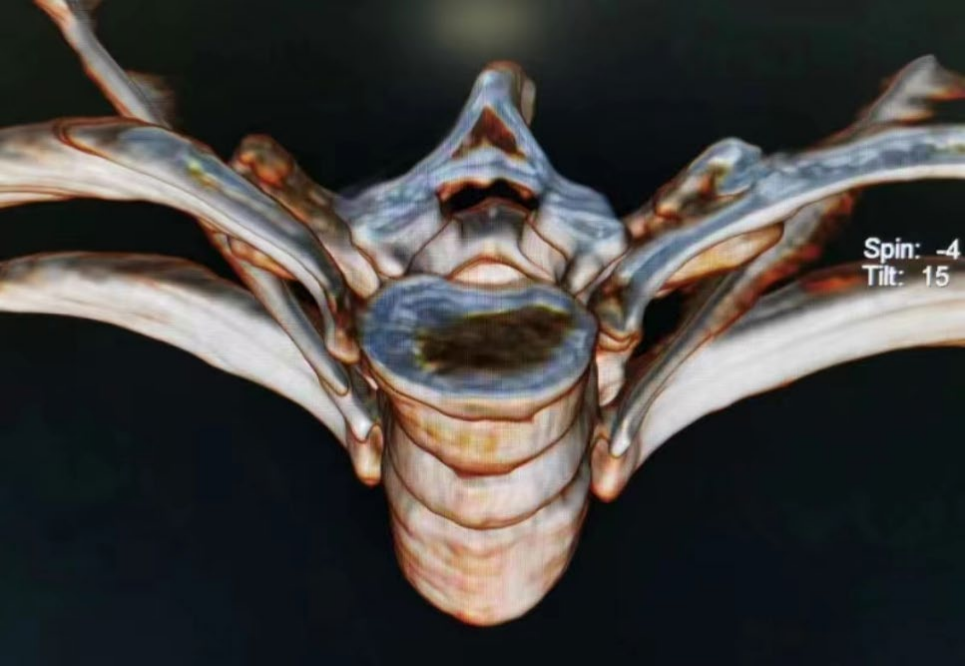
Radiofrequency thermocoagulation of bilateral thoracic sympathetic nerve(T4 Three-dimensional reconstruction
  However, T1-2 puncture is difficult to perform and can cause compensatory hyperhidrosis. The tip of the puncture needle is located on the outer side of the small head of the rib and close to the pleura, which carries the risk of pneumothorax and bleeding. Our specially designed blunt puncture needle reduces the risk of complications. The bending needle technique can help us smoothly cross the costotransverse joint and place the puncture needle in the target position (Fig. 7).

**Fig. 7:**
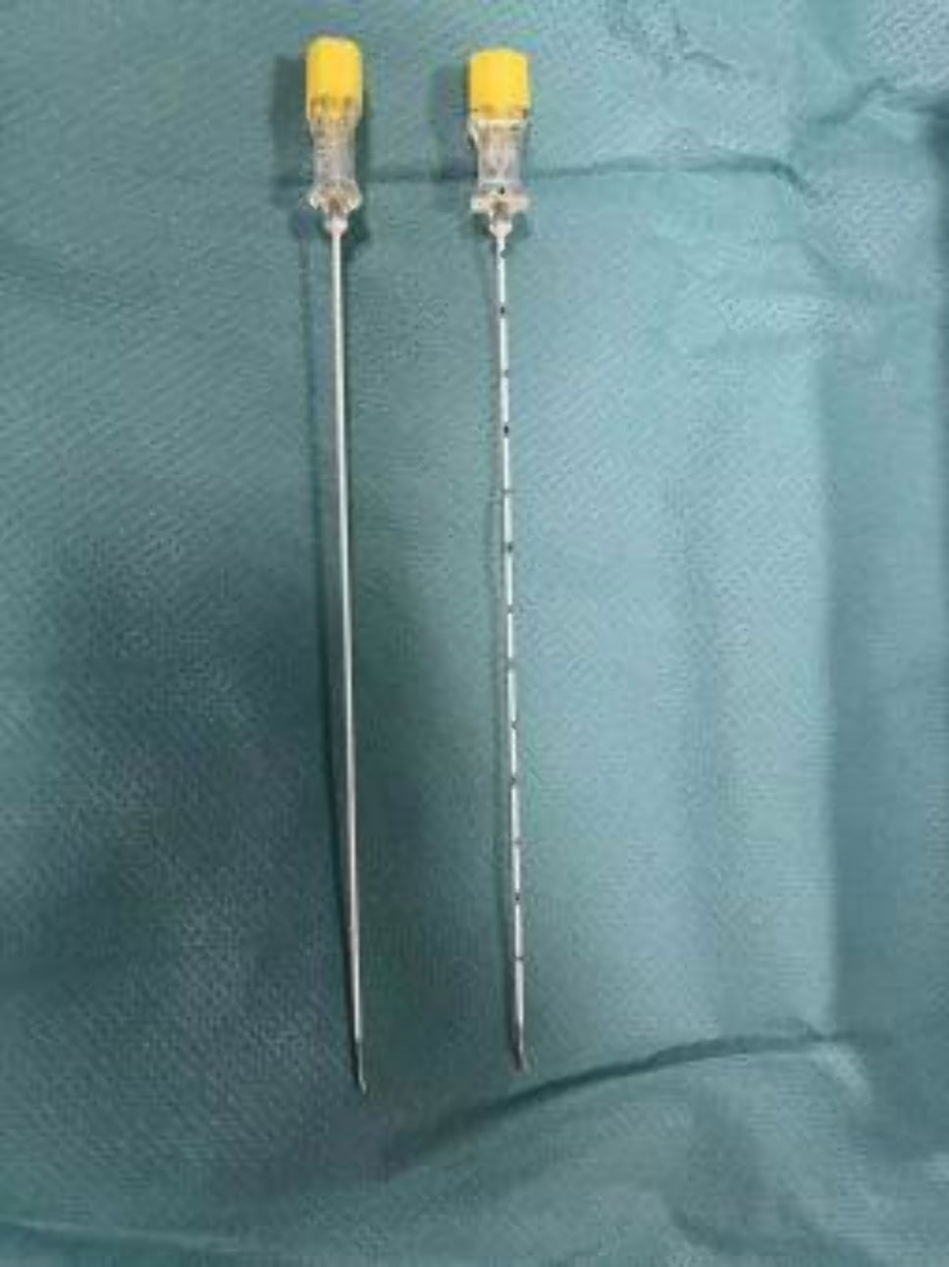
Specially made blunt puncture needle
6. Radiofrequency thermocoagulation of lumbar sympathetic nerve
  Lumbar sympathetic nerve radio-frequency (RF) technology is relatively mature. Taking the L3 sympathetic nerve RF as an example, the surgical procedure was as follows: The patient was placed in a prone position, the third lumbar vertebra was scanned, and the layer thickness was set to 2 mm. The appropriate injection path, angle, and depth were chosen to avoid nerves, blood vessels, and organs. Metal marks are made on the skin surface of the human vertebral body during CT scanning. The skin was disinfected, covered, and administered local anesthesia. The puncture was performed slowly using a radiofrequency electrode trocar. The CT scan confirmed that the needle tip reached the target position, namely the anterolateral edge of the L3 vertebral body and the front of the psoas major muscle (Fig. 8). If the patient does not have radiating pain in the lower extremity or groin area and has a sense of swelling in the waist, there is no sensory nerve in the damaged area. After motor nerve stimulation at 1 V and 2 Hz, there were no convulsions in the muscles of the lower extremities or buttocks and there were no motor nerves in the damaged area. The puncture needle is gradually warmed from 42 ℃ to 95 ℃ for 300 s of thermocoagulation treatment, after which the needle is withdrawn. The patient was returned to the ward when vital signs were stable.

**Fig. 8:**
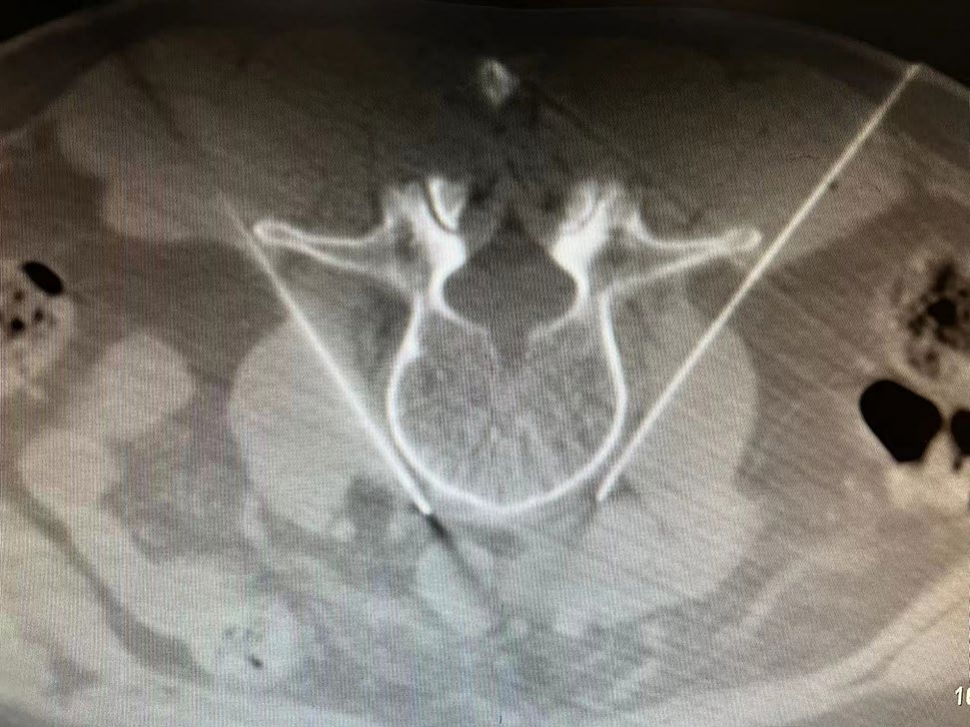
Radiofrequency thermocoagulation of bilateral lumbar sympathetic nerve
  Studies have shown [57–59] that CT-guided lower back sympathetic RF thermocoagulation has been gradually applied to the treatment of many autonomic nerve diseases, such as plantar hyperhidrosis (PH), cold hypersensitivity (CH), and diabetic peripheral neuropathy (DPN). Another study reported [60, 61] that lumbar sympathetic radiofrequency thermocoagulation achieved a lasting effect in the treatment of CRPS, with a pain relief rate of 91.7% at three months and 83.3% at six months after treatment. Radiofrequency thermocoagulation of the lumbar sympathetic nerve has a lasting and reliable effect on neuropathic pain in the lower extremities. It can improve blood supply to the lower extremities and relieve ischemia and pain symptoms to a certain extent.
  Unlike traditional sympathetic nerve block and chemical neuroregulation, radiofrequency thermocoagulation of the thoracic and lumbar sympathetic nervous systems locates the target nerve through electrical stimulation and uses high temperatures for thermocoagulation [62]. This process causes proteins to denature and coagulate, thereby blocking the transmission of nerve impulses. This method has the advantages of a precise destruction range, minimal damage, lasting efficacy, and high safety, and has been widely used by clinicians and patients.
7. Dorsal root ganglion pulse radiofrequency combined with sympathetic radiofrequency ablation
  Radiofrequency ablation increases blood perfusion in the skin and muscles by reducing sympathetic nerve tension and increasing collateral and peripheral blood volumes. Pain stimulation cannot be transmitted through nerve fibers to the central nervous system, thereby inhibiting pain transmission and achieving the purpose of reducing neuropathic pain. PRF plays an analgesic role by regulating disturbed electrical signals around the diseased dorsal root ganglia and nerve fibers, downregulating substance P levels in the dorsal root ganglia, and upregulating substance P levels in the spinal cord. Sympathetic radiofrequency can effectively control intractable neuropathic pain, such as PHN, CRPS, and PRF, through different mechanisms of action. The combined application of these two methods for the treatment of intractable neuropathic pain warrants further exploration and research in clinical practice.

## Discussion

Ultrasound-guided stellate ganglion block can treat trigeminal neuralgia, chronic migraine, and herpes zoster neuralgia with a good curative effect; however, its maintenance time is short, requiring repeated puncture injection therapy. Sympathectomy can completely remove the sympathetic nerve from the target segment; however, severe trauma and compensatory hyperhidrosis are complications. Chemical thoracic and lumbar sympathetic blocks have demonstrated significant efficacy in the treatment of upper- and lower-limb neuropathic pain. However, anhydrous alcohol as a chemical nerve-destroying agent has limitations in controllability and may cause damage to adjacent nerve tissue and even the spinal cord, thereby increasing the risk of complications. CT-guided RF thermocoagulation of the sympathetic nerve can achieve an accurate point-to-point treatment, which has the advantages of lasting efficacy, safety, accurate damage range, and effectively reduces pain in patients. Although sympathetic regulation has good clinical prospects for the treatment of neuropathic pain, certain challenges and unresolved problems remain. For example, the mechanism of sympathetic nerve regulation therapy is still not fully understood, and its effect on neuropathic pain requires further investigation. Previous studies have shown that sympathetic regulation plays a therapeutic role by inhibiting sympathetic nerve activity, reducing the production and release of pain-related mediators, and inhibiting pain transmission. However, the specific details and interrelationships among these mechanisms require further study.

## Conclusion

Sympathetic regulation can effectively relieve pain and improve the quality of life of patients by inhibiting sympathetic nerve activity, reducing the production and release of pain-related mediators, and inhibiting pain transmission. CT-guided sympathetic RF thermocoagulation technology can achieve accurate point-to-point treatment with significant and lasting effects and few complications and is recommended as a treatment for intractable neuropathic pain. Further research is needed on the efficacy and mechanisms of sympathetic regulation to address current challenges in the treatment of neuropathic pain.

## Data Availability

No Datasets were generated or analysed during the current study.
